# Salivary gland tissues and derived primary and metastatic neoplasms: unusual pitfalls in the work-up of sellar lesions. A systematic review

**DOI:** 10.1007/s40618-021-01577-6

**Published:** 2021-05-03

**Authors:** T. Feola, F. Gianno, M. De Angelis, C. Colonnese, V. Esposito, F. Giangaspero, M.-L. Jaffrain-Rea

**Affiliations:** 1 Neuromed Institute, IRCCS, Pozzilli, IS Italy; 2 Department of Experimental Medicine, University “La Sapienza”, Rome, RM Italy; 3 Department of Radiological, Oncological and Pathological Sciences, University “La Sapienza”, Rome, RM Italy; 4 Department of Neurology and Psychiatry, University “La Sapienza”, Rome, RM Italy; 5 Department of Biotechnological and Applied Clinical Sciences, University of L’Aquila, Via Vetoio, Coppito 2, 67100 L’Aquila, AQ Italy

**Keywords:** Ectopic salivary gland, Salivary neoplasm, Pituitary neoplasms, Sellar, Parasellar lesions

## Abstract

**Purpose:**

Salivary gland (SG) tissue and derived neoplasms may occur in the sellar region. As the current literature is mostly limited to case reports, the puzzling case of an inflammatory SG removed by transsphenoidal surgery (TS) and mimicking a prolactinoma prompted us to perform the first systematic review of these unusual conditions.

**Methods:**

A systematic literature search was conducted according to the PRISMA guidelines. Forty-four individual cases—non-neoplastic enlarged salivary glands (NNESG, *n* = 15), primary benign (*n* = 7) and malignant (*n* = 8) ectopic salivary tumours (ST) and sellar metastasis from eutopic primary ST (*n* = 14)—were suitable for the analysis of clinical, radiological and pathological characteristics. Therapeutic outcome was reviewed as a secondary endpoint.

**Results:**

All cases were diagnosed after surgery. NNESG commonly affected young and/or female patients, typically leading to headaches and hyperprolactinemia and originating close to the neurohypophysis. Submucosal SG should be excluded before concluding to an intrasellar NNESG after TS. No gender or age predominance was found for primary ectopic ST, which present as large tumors, with histological phenotypes similar to common ST. Hypopituitarism and diabetes insipidus were more frequent in ST than in NNESG. NNESG and benign ectopic ST rarely recur. Malignant ectopic ST should be distinguished from secondary localizations of eutopic ST reaching the sella by contiguity or metastatic spread; both share a frequent unfavorable outcome.

**Conclusion:**

Sellar neoplasms derived from SG are rare but misleading conditions and pituitary dysfunction is likely to be more common than currently reported. Appropriate pathological evaluation and multidisciplinary approach are required.

**Supplementary Information:**

The online version contains supplementary material available at 10.1007/s40618-021-01577-6.

## Introduction

Ectopic salivary gland (SG) tissue may occur in different sites of the body: extra-cranially (larynx, gastrointestinal tract, middle ear, chest wall) [1–6] and intra-cranially, with sellar and extra-sellar localizations (e.g.: optic nerve sheath, cerebellopontine angle) [7–9]. Intrasellar ectopic SG rests are typically localized close to the neurohypophysis or in the *pars intermedia*, often communicating with the Rathke’s cleft [7], and maybe incidentally found at autopsy [10, 11]. Only a small minority come to clinical attention because of mass effects and/or endocrine dysfunction, in particular hyperprolactinemia [8, 12–20]. Symptomatic enlargement of ectopic SG rests may be non-neoplastic (NNESG) or due to benign or malignant salivary tumours (ST) that mimic other non-functioning lesions, and the diagnosis relies on pathology where surgery is indicated. In addition, because malignant ST derived from major or minor eutopic SG may reach the sella through local invasion or blood spread [21–23], an extra-sellar origin should be excluded before concluding to a primary ectopic SG malignancy [24]. Sellar salivary neoplasms represent an unusual challenge for specialists involved in the management of pituitary neuroendocrine tumours (Pit-NETs) [25] and other sellar/parasellar lesions.

**Fig. 1:**
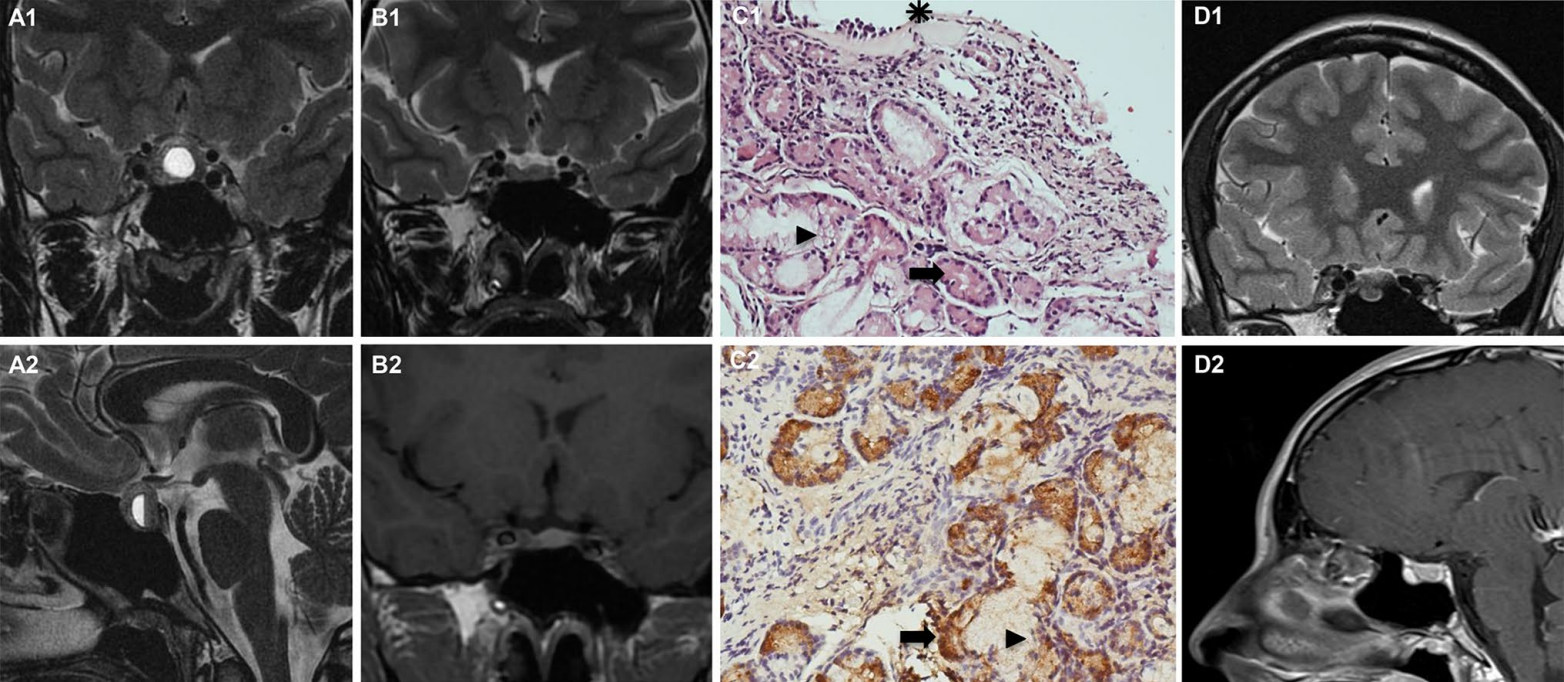
A puzzling case of sellar salivary gland (SG). A 19-year-old woman was referred in February 2019 because of a prolactinoma showing increasing pharmacological resistance. The diagnosis was made 3 years earlier in the setting of primary amenorrhea–galactorrhea and intermittent headache, plasma PRL 1763 ng/ml (*N* < 26.7) and a macroadenoma with a fluid hemorrhagic component at Magnetic Resonance Imaging (MRI) (**A1**, **A2** coronal and sagittal T2-weighted). Menarche occurred within 5 months of treatment, with regular menses but an increasing and poorly tolerated drug requirement to obtain a sub-optimal control of hyperprolactinemia (CAB up to 3.5 mg/week). As MRI showed clear evidence of residual disease (**B1**, **B2** pre-and post-Gadolinium coronal views), endoscopic TS was proposed. A small nodular lesion, consistent with a microadenoma, was removed. Unexpectedly*,* pathological examination revealed numerous groups of glandular berries composed of typical serous and mucinous cells, compatible with SG tissue, separated by a chronic inflammatory lymphoplasmacellular infiltrate (**C1** hematoxylin–eosin). Immunostaining for lysozyme was positive in mucinous cells (**C2**). Bony spicules and flaps of respiratory mucosae were also present, with no evidence of pituitary cells. Immunostaining for PRL was negative (not shown). The first pathological diagnosis was NNESG. However, post-operative CAB withdrawal was followed by a progressive recurrence of symptomatic hyperprolactinemia (up to 245 ng/ml 4 months after surgery), with MRI evidence of residual/recurring disease. Careful revision of serial pre-operative imaging revealed in a single MRI study (2017) a small intrasphenoidal nodular lesion localized just beneath the adenomatous lesion, with spontaneous hypointensity in T2 (**D1** coronal view) and hyperintensity in T1 before and after gadolinium (**D2** sagittal view). This finding was consistent with a cystic SG, undergoing subsequent inflammation and shrinkage. The final diagnosis was a sub-mucosal SG, mimicking and masking the residual microprolactinoma during TS. As CAB was re-started up to the maximal well-tolerated dose (2.0 mg weekly) with an incomplete response (PRL 45 ng/ml), TS will be potentially re-considered if necessary.

A recent puzzling observation (illustrated in Fig. 1) prompted us to perform the first systematic review of the literature about sellar NNESG and ST, pointing out an additional diagnostic pitfall, i.e. an inflammatory submucosal SG mimicking a prolactinoma during transsphenoidal surgery (TS). Individual cases were classified into four groups: ectopic NNESG, benign and malignant ectopic ST (eST) and secondary localizations of eutopic ST. Clinical, neuroradiological, pathological characteristics, and therapeutic outcome were analysed. This review points out the importance of a multidisciplinary work-up to reach a correct diagnosis and optimize clinical management.

## Methods

A systematic review of case reports and case series was performed according to the Cochrane Collaboration and PRISMA statements [26, 27]. A literature search without limits was conducted on Medline and Scopus up to September 2020, including international and non-English literature, using the following keywords: *ectopic salivary gland/ salivary gland/salivary gland tumour* AND *pituitary/sella/sphenoidal/sphenoid sinus*. Cross-references were used to identify additional papers, allowing to retrieve six additional cases. Titles and abstracts of all papers were screened to assess their relevance. Duplicates, reviews, animal studies, in vitro studies and congress reports were excluded. Based on available abstracts and full texts, all the papers describing NNESG and benign or malignant sellar ST were analyzed. The following data were extracted for each paper: (1) first author, year of publication; (2) case demographics (gender, age), (3) symptoms, (4) endocrine abnormalities, in particular, PRL values, (5) neuro-radiological findings at MRI and/or computed tomography (CT): localization, size, invasion, signal intensity/density, contrast enhancement, (6) pre-operative and final pathological diagnosis, (7) treatment and, (8) where available, status at last follow-up (recurrence, progression, hormone replacement therapy, death).

## Results

**Fig. 2:**
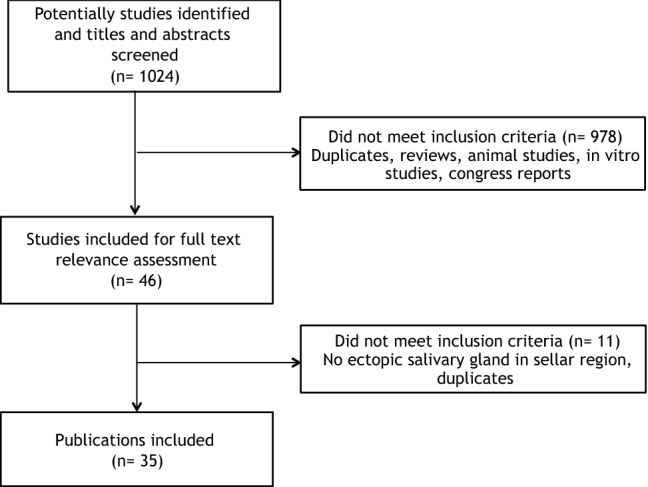
Flowchart of the literature eligibility assessment process

Overall, 1024 potentially relevant studies were found, 978 were excluded at first screening and 46 were selected for full-text assessment (Fig. 2). Thirty-five papers were finally retained (1963–2020): 32 in English language, 1 in French, and 2 papers in Japanese or Korean with detailed English abstracts and figures footnotes. Overall, 44 individual cases of symptomatic sellar NNESG and ST were described, including 14 secondary sellar localizations of primary eutopic ST. Because PitNETs were originally reported as pituitary adenomas (PA) in all papers, we elected to maintain this terminology to report the pre-operative diagnosis.

### NNESG

Fifteen cases of NNESG were found (Table 1) [8, 12–20, 28]. Most patients were younger than 30 years (11/15, 73.3%), including a pediatric case. Most were females (12/15, 80%). The most frequent complaints were headache (12/15, 80%), visual symptoms—bitemporal hemianopsia, blurred vision, decreased visual acuity—(4/15, 26.6%), nausea (4/15, 26.6%), galactorrhea and menstrual irregularities (3/15, 20%). Endocrine dysfunction was frequent (8/15, 53.3%), including mild hyperprolactinemia (23.9–93.0 ng/ml, median 83.5) (3/15, 20%), growth hormone deficiency (2/15, 13.3%), panhypopituitarism (2/15, 13.3%) and/or central hypothyroidism (1/15, 6.7%). Pre-operative diabetes insipidus (DI) was present in 3/15 cases (20%). NNESG were variable in size (maximal dimension 0.6–4.6 cm, median 1.7). Intrasellar lesions were typically localized in the posterior pituitary (6/15, 40%), suprasellar extension was frequent (8/15, 53.3%), but hydrocephalus was rare (1/15, 6.7%) [28]. Lateral extension was uncommon (2/15, 13.3%), with cavernous sinus infiltration in the largest case [16]. Based on the neuroradiological description and/or pre-operative diagnosis, they presented as pseudo-tumorous solid lesions, with frequent cystic component(s) (7/15, 46.7%). At MRI, most lesions appeared as hyper- or iso-intense on T1 weighted imaging (T1) (7/15, 46.7% and 4/15 26.6%, respectively) but either hyper- or hypo-intense on T2 weighted imaging (T2) (4/15, 26.6% and 4/15, 26.6%, respectively), with inconstant contrast enhancement (4/15, 26.6%). Spontaneous hyperdensity was found at CT where available (*n* = 4). Pre-operative diagnosis was: PA (8/15, 53.3%), Rathke’s cleft cyst (RCC) (5/15, 30%), craniopharyngioma (2/15, 13.3%), exceptionally chordoma (the largest one) [16]. Two patients received bromocriptine but the lesion was unchanged despite PRL normalization [12, 20]. All patients were operated on, 80% through a TS route (12/15). Follow-up was limited (1–4 years, median 1 year, *n* = 10), but no recurrence or progression was reported, except a cystic relapse after 2 years, without pathological evidence of SG tissue [28]. At pathological examination, SG rests or cysts were found within or close to the posterior pituitary lobe, in 7 cases within the wall of a RCC (46.7%). NNESG were composed of acini with a low columnar or cuboidal epithelium, embedded in a fibrovascular stroma, without cellular atypia, with occasional inflammation (2/15, 13.3%). Anti-PGP immunoreactive nerve fibers were reported in one case, suggesting parasympathetic innervation [18]. Adjacent anterior pituitary cells were observed in a minority of cases (20%).

**Table 1:** Non-neoplastic enlarged ectopic sellar salivary glands (NNESG) reported in the literature

Publication	Sex, age (years)	Symptoms	Endocrine dysfunction	Plasma PRL	Imaging (MRI/CT)	Size (cm)	First diagnosis	Treatment	Histopathological findings	Follow-up (duration)
Kato et al. [14]	M, 11	Growth retardation	GHD	NA	Sellar/suprasellar Posterior pituitary lobe Cystic Mildly hyperintense on T1 and T2 (MRI)	NA	NA	Surgery (TS)	Cyst in the posterior pituitary lobe Acid to neutral mucopolysaccharides content Acinar tissue with a simple epithelium formed of cuboidal or columnar cells	NA
Tatter et al. [20]	F, 22	Headache Galactorrhea Irregular menses	None	(23.9 ng/ml)	Sellar Posterior pituitary lobe Isointense on T1, iso-hypointense on T2, no CE (MRI)	1.2 × 0.9 × 0.9	PA	DA (2 months) then Surgery (TS)	Well-formed salivary acini with a low columnar epithelium in a fibrovascular stroma Eosinophilic content No anterior pituitary cells in the cyst lining	No recurrence, no HRT and normal PRL a (12 months)
Chen et al. [12]	F, 28	Headache Galactorrhea Irregular menses	None	(93 ng/ml)	Sellar Posterior pituitary Isointense on T1 and T2, no CE (MRI)	0.6 × 0.5	Cystic PA or RCC	DA (2 years) Surgery (TS)	Mixed nests of acidophilic, basophilic and chromophobic cells in a delicate fibrovascular network Colloid-like content Mild chronic inflammation	No recurrence, no HRT and normal PRL (12 months)
Kim et al. [15]	F, 19	Headache Blurred vision Nausea Dizziness	None	Normal	Sellar/suprasellar hyperintense on T1, with CE (MRI)	1.8	PA	Surgery (TS)	Cyst in the posterior pituitary Seromucinous acini with a low-columnar to cuboidal epithelium in a fibrovascular stroma No evidence of pituitary adenoma	Post operative DI
Ranucci et al. [17]	M, 17	Headache Nausea	NA	(83.5 ng/ml)	Sellar/suprasellar, contacting the medial CS walls bilaterally	1.9	NA	Surgery (TS)	Lobules of seromucous glands, embedded in a fibrovascular network, within the wall of a RCC Anterior pituitary tissue	Post-operative PRL 33.1 ng/ml
Stefanits et al. [18]	F, 23	Headache Galactorrhea Irregular menses	None	NA	Sellar Posterior cyst Hyperintense on T1, no CE	1.5^a^	PA, RCC	Surgery (TS)	Tubular glands with intraluminar mucous embedded in fibrous connective tissue and cystic cavities lined by non ciliated epithelium compatible with RCC Close to the anterior pituitary, no adenoma Anti-SMA immunoreactive epithelial cell and anti-PGP immunoreactive nerve fibers surrounding the lesion	HRT (L-T4, hydrocortisone, desmopressin)
Hwang et al. [13]	F, 26	Headache Nausea	None	NA	Sellar Posterior pituitary lobe Hyperintense in T1, hypointense in T2, no CE (MRI)	1.9 × 0.5 × 0.9	PA with apoplexy	Surgery (TS)	Cyst in the posterior pituitary Salivary acini with a low-columnar epithelium in a fibrovascular network Eosinophilic content No evidence of neoplasm	NA
Hintz et al. [8]	F, 28	Headache Decreased vision Bitemporal hemianopsia Insatiable appetite and weight gain Polyuria	NA	NA	Sellar/suprasellar Hypointense on T1, hyperintense on T2, no CE (MRI)	NA	NA	Surgery (TC)	Branching tubules and small glands or acini lined by attenuated to columnar epithelium Pale blue mucinous material content No secretory granules, atypia, or mitotic activity No respiratory or ciliated epithelium, no goblet cells	No recurrence (2 years)
Tanaka et al. [19]	M, 24	Headache Bitemporal Hemianopsia	Central HT		Sellar Cystic, hyperintense on T1, hypointense on T2, no CE	1.6	NA	Surgery (TS)	Mucopolysaccharide content Cyst wall surrounded by myoepithelial cells positive for P63 staining, no atypical cells Lymphocytic infiltration (acute inflammation), proliferation of macrophages, fibrosis, and foam cells (chronic sialadenitis)	No recurrence (12 months)
Liu et al. [16] Case 1	F, 57	Headache	None	Normal	Sellar/suprasellar surrounded bilateral internal carotid arteries Isointense on T1, iso-hyperintense on T2, heterogeneous CE Hyperdensity (CT)	4.6	Chordoma	Surgery (TS)	Lobules of seromucous glands Fragments of normal pituitary tissue No evidence of neoplasia	No progression (12 months)
Liu et al. [16] Case 2	F, 36	Headache	GHD (primary hypothyroid-dism)	Normal	Sellar Posterior pituitary lobe Hyperintense on T1, hypointense on T2, no CE (MRI) Hyperdensity (CT)	0.8 × 1.7 × 1.3	PA, RCC	Surgery (TS)	Scattered islands of seromucous glands mixed with fragments of simple columnar epithelium, constituting the lining of the RCC	No recurrence (4 years)
Liu et al. [16] Case 3	F, 48	Nausea Blurred visive	None	NA	Sellar/suprasellar Isointense on T1, hyperintense on T2, Rim CE (MRI) Hyperdensity (CT)	1.7	PA	Surgery (TS)	Scattered islands of seromucous glands mixed with fragments of squamous and ciliated columnar epithelium, constituting the lining of the RCC	No recurrence (12 months)
Kleinschmidt-DeMasters et al. [28] Case 1	F, 22	Hydrocephalus	Panhypopit DI	NA	Sellar/suprasellar Heterogeneous signal on T2, peripheral CE on T1 Third ventriculomegaly	2.4 × 2.2 × 2.4	CP, RCC	Surgery (TS)	Cystic sellar salivary gland 90% Acellular amorphous eosinophilc cyst contents typical of RCC with low cuboidal ciliated epithelium 10% acinar glands lined by low cuboidal epithelium without stroma or inflammation, focally showed globet cells as the source of mucin No cytological atypia, mitosis or necrosis	HRT, Cyst recurrence after 2 years (without salivary gland like tissue)
Kleinschmidt-DeMasters et al. [28] Case 2	F, 29	Headache	NA	NA	Sellar Cystic and solid components	NA	NA	Surgery (excisional biopsies)	Cystic salivary gland in the posterior pituitary lobe Salivary glands producing mucin without cytological atypia or mitosis, focally showing eosinophilic cytoplasma reflecting oncocytic change Amorphous eosinophilc colloid material lined by low cuboidal cells identical to RCC	< 12 months
Kleinschmidt-DeMasters et al. [28] Case 3	F, 68	Headache Fatigue	Panhypopit DI	NA	Sellar/suprasellar Hyperintese on T1	1.3 × 1.4 × 1.9	PA with apoplexy, CP, RCC	Surgery excisional biopsies	Salivary-type glands adjacent to a thin fibrotic cyst wall Amorphous eosinophilc mucin Ciliated columnar epithelium identical to RCC	< 12 months

*CE* contrast enhancement, *CT* computed tomography, *CP* craniopharyngioma, *DA* dopamine-agonist, *DI* diabetes insipidus, *GHD* growth hormone deficiency, *HRT* hormone replacement therapy, *MRI* magnetic resonance imaging, *NA* not available, *Panhypopit*. panhypopituitarism, *PA* pituitary adenoma, *PGP* protein gene product, *PRL* prolactin, *RCC* Rathke’s Cleft Cyst, *SMA* smooth muscle actin, *T1* T1-weighted imaging, *T2* T2-weighted imaging, *TC* transcranial, *TS* transsphenoidal

^a^Slowly progressive growth

### Sellar ST

The individual characteristics of sellar ST according to their pathological classification are shown in Table 2.

**Table 2:** Sellar/parasellar salivary tumours (ST) reported in literature

Publication	Sex, age (years)	Symptoms	Endocrine dysfunction	Plasma PRL	Imaging (MRI/CT)	Size (cm)	First diagnosis	Treatment	Histopathological findings	Follow-up (duration)
**(I) Primary benign ectopic ST**										
Hampton et al. [29] Case 1	F, 61	Nausea, vomiting Occasional hypothermia Decreased visual acuity Bitemporal hemianopsia	NA	NA	Sellar/suprasellar (optic chiasm)	3.0 × 3.5	PA	Surgery (TS) RT (66 Gy)	Pleomorphic adenoma (Ki67 12.9%)	No recurrence (24 months)
Hampton et al. [29] Case 2	F, 81	Dehydration Anorexia Mental status changes Bitemporal hemianopsia	ACTH, TSH deficit		Sellar/suprasellar 3° ventricle and hypothalamus compression	NA	PA	Surgery (TS) RT (65 Gy)	Monomorphic adenoma (Ki-67 2.36%)	NA
Hampton et al. [29] Case 4	F, 17	Headache Confusion Homonymous hemianopsia	Panhypopit., DI	NA	Sellar/suprasellar CE (MRI) Hemorrhagic mass (CT)	NA	PA	Surgery (TC and TS)	Salivary gland oncocytoma (Ki67 0.6%)	No recurrence (3 months) HRT
Chimelli et al. [7]	M, 44	Headaches Weakness Impotence, decreased libido Decreased vision Bitemporal hemianopsia	Panhypopit	Normal	Sellar/suprasellar (optic chiasm)	2.5 × 2.5 × 0.5	PA	Surgery (TS)	Pleomorphic adenoma within the wall of a RCC	No recurrence (15 months) HRT
Rychly et al. [30]	M, 38	Muscle weakness Axillary hair loss Reduction of perspiration Weight loss Progressive visual and mobility loss	NA	NA	Sellar/suprasellar (optic chiasm) Heterogeneous CE (MRI)	3.0 × 3.5 × 4.0	CP	1° surgery (TC) RT (60 Gy) 2° surgery (TS)	Adenomyoepithelioma (Ki67% 5%—recurrence vs. 0%—original tumor)	Recurrence after 1° surgery (14 years) No recurrence since 2°surgery (6 months) HRT, DI
Takahashi et al. [31]	M, 56	Thirst Fatigue Decreased visual acuity	DI	NA	Sellar/suprasellar Hypothalamus and midbrain compression Well-defined borders and heterogeneous CE (MRI) Calcifications (CT)	NA	Chordoma	1° surgery RT 2° surgery (TC)	Pleomorphic adenoma	No recurrence (3 years)
Yao et al. [32]	M, 23	Polyuria, polydipsia Asymmetrical breast development Fever Visual loss	ACTH, LH, FSH deficit and DI		Sellar/suprasellar (optic chiasm)	NA	PA, benign teratoma	1° surgery (TC) (subtotal) 2° surgery (TC) (radical)	Pleomorphic adenoma (Ki67 1.2%)	Recurrence after first surgery (4 years) No recurrence since 2nd surgery (30 months)
**(II) Primary malignant ectopic ST**										
Hampton et al. [29] Case 3	F, 66	Bilateral VI nerve palsy	NA	NA	Sellar/suprasellar Inferior invasion and bilateral CS extension encasing the carotids	NA	PA	Surgery (TC) RT (50 Gy) Surgery for meningeal metastasis	Adenocarcinoma, low grade (Ki67 13% primary, 15% met.)	Secondary intracranial dissemination (4 years)
Gilcrease et al. [33] Case 1	F, 44	Galactorrhea Amenorrhea Hemianopsia	NA		Sellar/suprasellar (optic chiasm)	3.8	ACC (biopsy)	Surgery	ACC adjacent to RC epithelium	Post-operative death (8th day, severe hypotension)
Gilcrease et al. [33] Case 2	M, 55	Diplopia	NA	NA	Sellar/suprasellar	2	NA	Surgery (TS)	Papillary mucinous adenocarcinoma adjacent to RC epithelium	No recurrence Alive (5 years)
Tsuyuguchi et al. [36]	F, 34	Galactorrhea Amenorrhea Visual loss	Panhypopit	(65.2 ng/ml)	Sellar/suprasellar Cystic area on T2, with CE (MRI) No bone erosion (CT)	NA	CP	Surgery (TC) RT (20 Gy) *10 radiosurgeries and 4 operations*	ACC (Ki67: 11%)	Recurrences at 3 and 7 months Death (3 years)
Nieder et al. [35]	F, 34	Bitemporal hemianopsia Visual impairment	Panhypopit., partial DI	NA	Sellar/suprasellar (hypothalamic compression)	NA	CP	Surgery (subtotal resection, cerebral met.) RT (54 Gy) CHT (isofosfamide and BCNU)	Malignant myoepitelioma (Ki67 30–40%)	Tumour progression Death (20 months)
Van Furth et al. [37]	M, 60	Anorexia Fatigue Headache Addisonian crisis Partial III nerve palsy	ACTH deficit, DI	NA	Sellar/suprasellar (floor of the 3° ventricle) Homogenous CE (MRI)	2.5 × 1.8 × 2	PA	Surgery (TS)	Acinic cell carcinoma, low grade (Ki67 3%)	Post-operative death (8th day, rupture of thoracic aortic aneurysm)
Hong et al. [34]	F, 48	Left III and VI nerve palsy Sensory symptoms on the left forehead skin	None	NA	Left CS mass Extension into the ipsilateral middle fossa Isointense on T1, hypointense on T2, intense and heterogenous CE (MRI)	NA	PA, granulomatosis, metastatic brain tumor, primary of CS	Surgery (TC)	Malignant myoepitelioma (CS) (Ki67 60%)	Residual tumour regrowth Death (2 weeks after second surgery)
Lavin et al. [24]	M, 68	Weight loss Reduced muscle bulk Confusion and drowsiness for obstructive hydrocephalus Partial III nerve palsy	Panhypopit	(451 mU/l)	Sellar/suprasellar (floor of the 3° ventricle) Left CS Solid mass Cystic component, heterogeneous CE (MRI)	NA	NA	Surgery (TC) RT (54 Gy) Temozolomide	EMC (Ki67 40%)	Tumour progression Death (22 months, pulmonary infection)
**(III) Secondary malignant ST in the sellar region**										
Taillens et al. [43]	M, 52	Diplopia VI nerve palsy Multiple cranial nerve palsies Visual loss Headache Weight loss	NA	NA	Sellar/suprasellar (optic chiasm compression) CS invasion Skull base erosion (X-ray)	NA	PA	RT Surgery (TS)	Mixed salivary tumor Nasopharynx Pituitary and intracranial invasion	HRT Post-operative death 15 days, meningitis)
Vincentelli et al. [45] Case 1	F, 35	Diplopia Blindness VI nerve palsy Orbital pain Hearing loss	NA	NA	Sellar/suprasellar Enlargement of optic canal Bone erosion (sellar) (X-ray)	NA	Neurinoma, meningioma	Surgery RT (60 Gy)	ACC Local invasion (SS)	Recurrence (4 years) Death
Vincentelli et al. [45] Case 4	F, 29	Headache Amenorrhea Galactorrhea	NA	NA	Round mass filling the SS Bone erosion (sellar floor and clivus)	NA	NA	Surgery (TS) RT (70 Gy) *Re-operations*	ACC Local invasion (SS) Recurrent (delay: 5 years)	Recurrence (2 years) Two re-operations Death
Dickhoff et al. [38]	F, 41	VI nerve palsy	None		NA	NA	NA	NA	ACC Local invasion (SS)	NA
Hampton [29] Case 5	F, 85	NA	NA	NA	Skull base destruction (CT)	NA	PA	Surgery (TS)	Monomorphic adenoma Parotid Multi-recurrent Direct extension	Death (11 years)
Kaur et al. [23]	M, 33	NA	NA	NA	Sellar CS invasion Anterior right temporal lobe (CT)	NA	NA	Surgery (TC)	ACC Right palate Recurrence (delay:12 years) Dural invasion	NA
McCutcheon et al. [42]	M, 47	Polyuria Polydipsia Weight loss Cold and heat intolerance Decreased energy and libido Mild diplopia Bitemporal hemianopsia	Panhypopit., DI		Sellar/suprasellar (optic chiasm) Posterior extension Isointense on T2, heterogeneous CE (MRI) Posterior peri-tumorous edema	NA	NA	Surgery (TC) RT (30 Gy)	Ductal adenocarcinoma Parotid Metastasis	Death (7 months)
Kawamata et al. [40]	F, 78	General malaise Disturbed consciousness Hyponatremia	SIADH	(26.2 ng/ml)	Sellar/suprasellar (optic chiasm) Suspect intra-tumorous hemorrhage Partial CE (MRI)	NA	Met. with intratumoral hemorrhage, CP, PA with apoplexy	Surgery (TS) RT	ACC Parotid Metastasis (delay: 4 years) (Ki67 12.5%)	NA
Abdul-Hussei [39]	F, 49	Headache Photophobia Dizziness Nausea Diplopia VI nerve palsy Numbness in the right V area (complete)	None	(39.1 ng/ml)	Large mass in the clivus with posterior destruction, destruction of the pterygoid palate and anterior extension (SS and nasopharynx) Right CS invasion Bone invasion (CT)	3.8 × 3 × 2	NA	Surgery (TC) RT	ACC Local invasion (SS)	NA
Tripathy et al. [44]	M, 28	Bloody nasal discharge Nasal blockage Blurred vision Hemianopsia	NA	NA	Mass in the left ethmoid and SS extending to the sella, nasal cavity and nasopharynx Hypointense in T1, hyperintense in T2, heterogeneous CE (MRI)	NA	NA	Surgery (TS) RT	ACC Paranasal sinus Local invasion	No recurrence or metastasis (6 months) Small residual lesion (CS)
Giridhar et al. [21]	M, 38	Headache Sensory loss—maxillary division of V nerve Diplopia	NA	NA	Mass in the left SS Bone erosion (skull base, SS, sphenoid and left petrous apex), bilateral CS and left orbital apex (CT)	NA	NA	RT (66 Gy)	ACC Local invasion (SS)	No progression (6 months) Symptoms resolution
Kenan et al. [41]	F, 43	Headache Vision loss in the right eye	NA	NA	Mass in the left paraclinoid area, adjacent to the optical nerve	1.6 × 1.2	NA	Surgery (TC)	ACC Nasopharynx Metastasis (perivascular route) (delay: 3 months)	No complications
Hughes et al. [22]	F, 72	Fall and facial trauma Polyuria Incontinence Bitemporal hemianopsia	TSH, ACTH deficit	NA	Sellar/suprasellar (optic chiasm, involvement of anterior cerebral arteries) CE (MRI) Sellar bone destruction (CT)	3.8 × 2.3 × 2.1	PA	Surgery (TS) RT (37 Gy)	ACC Parotid Metastasis (delay: 26 years)	HRT
Jahandideh et al. [46]	M, 69	Headache Diplopia Sensory loss in the V nerve area	NA	NA	Mass in the right SS extending to the sella and clivus Hypointense on T1 and hyperintense on T2, CE (MRI)	4 × 2 × 2	ACC, chordoma	Surgery (endoscopic) CHT-RT	ACC Local invasion (SS)	Delayed post-operative death (3 months, respiratory distress)

Italic text inside refers to multiple treatments received for tumour regrowth

*ACC* adenoid cystic carcinoma, *CE* contrast enhancement, *CHT* chemotherapy, *CP* craniopharyngioma, *CS* cavernous sinus, *CT* computed tomography, *DI* diabetes insipidus, *GHD* growth hormone deficiency, *HRT* hormone replacement therapy, *MRI* magnetic resonance imaging, *NA* not available, *Panhypopit*. Panhypopituitarism, *PA* pituitary adenoma, *PRL* prolactin, *RC* Rathke’s cleft remnants, *RCC* Rathke’s cleft cyst, *RT* radiotherapy, *SS* sphenoid sinus, *T1* T1-weighted imaging, *T2* T2-weighted imaging, *TC* transcranial, *TS* transsphenoidal

^a^No full text available

### Primary benign ectopic ST

Seven cases of benign sellar eST have been reported [7, 29–32], with a majority of pleomorphic adenomas (4/7, 57.1%) and single reports of monomorphic adenoma, oncocytoma and adenomyoepithelioma. They manifested at any age in both genders (4M, 3F, 17–81 years-old, median 44), with visual symptoms in all cases and inconstant headache (2/7, 28.6%). General symptoms—such as muscle weakness, fatigue, weight loss, anorexia, nausea and/or vomiting—were also present in all patients, and endocrine dysfunction reported as hypopituitarism (4/7, 57.1%), hyperprolactinemia (2/7, 28.6%) and/or DI (3/7, 42.8%). Accordingly, benign eST were large (2.5–4.0 cm, median 3.0), with a suprasellar extension in all cases—up to the optic chiasm (5/7, 71.4%) or the hypothalamus (2/7, 28.6%). MRI signal was not reported, but contrast enhancement was frequent (3/7, 42.8%) and calcifications or pseudo-hemorrhage could be found at CT. The most frequent pre-operative diagnosis was PA (*n* = 5), but craniopharyngioma, chordoma and benign teratoma were also considered. All patients were operated on—TS (4/7, 57.1%) or transcranially (TC) (3/7, 42.8%). Based on a variable follow-up duration (0.4–14 years, median 2.5, *n* = 6) recurrences were rarely reported after complete surgical removal, but occurred 4 and 14 years after partial removal [29–31]. Four patients received radiotherapy [29–31]. Noteworthy, the first pathological diagnosis was inaccurate in 3 cases (benign teratoma, chordoma, craniopharyngioma) [30–32].

#### Primary malignant ectopic ST

Primary malignant eST were reported in 8 cases [24, 29, 33–37] and consisted of adenoid cystic carcinoma (2/8, 25%), myoepithelioma (2/8, 25%), epithelial–myoepithelial carcinoma (1/8, 12.5%), papillary mucinous adenocarcinoma (1/8, 12.5%), low-grade acinic cell carcinoma (1/8, 12.5%) and adenocarcinoma (1/8, 12.5%). All patients were adult (3M, 5F, 34–68 years, median 51.5) and had visual symptoms, with frequent oculomotor nerve palsy/diplopia (5/8, 62.5%). Endocrine dysfunction was reported as hypopituitarism (4/8, 50%)—including one case of acute adrenal insufficiency, hyperprolactinemia (3/8, 37.5%) and DI (2/8, 25%). Accordingly, tumours were large (2.0–3.8 cm, median 2.5), growing up to the optic chiasm (4/8, 50%) or the floor of the 3° ventricle (3/8, 37.5%). An invasive growth was reported in some cases, eroding inferiorly into the sphenoid and ethmoid sinuses (1/8, 12.5%) or extending in the cavernous sinus (2/8, 25%)—in one case reaching the middle fossa [34]. Contrast enhancement was inconstantly reported (4/8, 50%) and, according to limited detailed MRI (*n* = 2), the tumour was isointense on T1 and slightly hyper- or hypointense on T2 [34, 36]. Pre-operative diagnosis was PA (*n* = 3) [29, 34, 37], craniopharyngioma (*n* = 2) [35, 36], but also inflammatory granulomatosis/hypophysitis, metastatic brain tumour and primary tumour of the cavernous sinus [34]. All patients were operated on—half of them through a TC approach—and subsequently irradiated (20–54 Gy). Two patients also received chemotherapy (isofosfamide/BCNU or temozolomide) for an epithelial–myoepithelial carcinoma and an aggressive myoepithelioma, respectively [24, 35], with a poor response. Except for one case of papillary mucinous carcinoma [33], all patients showed disease progression within 4 years (4/8, 50%) or died (6/8, 75%, 3 post-operative deaths). In one case, neuropathological misdiagnosis of PA was reported [29].

#### Secondary malignant sellar ST

Secondary ST were reported in 14 patients, deriving in most cases from minor salivary glands situated in the sphenoid sinus (*n* = 6), nasopharynx (*n* = 2), palate (*n* = 1) and paranasal sinus (*n* = 1)—but also from the parotid gland (4/14, 28.6%) [21–23, 29, 38–46]. A majority were adenoid cystic carcinoma (ACC) (11/14, 78.6%), with single reports of ductal adenocarcinoma, mixed salivary tumour, and a monomorphic multi-recurrent parotid adenoma extending to the sella. Most primary ST reached the sella through the sphenoid sinus (9/14, 64.3%) or dural infiltration (1/14, 7.1%), but metastatic blood spread could occur (4/14, 28.6%). In 4 cases sellar involvement was delayed (5–26 years after the primary tumour) [22, 23, 40, 45]. Adults were affected at any age (6M, 8F, 28–85 years, median 45). Most patients had visual defects (10/14, 71.4%), cranial nerve palsy (7/14, 50%) with frequent diplopia (6/14, 42.8%) and/or trigeminal sensory symptoms (3/14, 21.4%). Endocrine dysfunction (hypopituitarism, hyperprolactinemia, DI, syndrome of inappropriate antidiuretic hormone secretion—SIADH) was documented in a minority of cases (5/14, 35%) despite suggestive symptoms in additional cases. Tumours were large, frequently invasive with skull base erosion (6/14, 42.8%) or cavernous sinus infiltration (5/14, 35.7%)., and inconstant suprasellar invasion (5/14, 35.7%). Pre-operative diagnosis was PA (*n* = 3) [22, 29, 43]—with an apoplectic presentation in one case [40]—but also craniopharyngioma, meningioma, neurinoma and metastasis [40, 45]. Surgery was proposed in all but one patient, who received first-line radiotherapy for tumour inoperability [21]. One patient who initially declined surgery was operated on 5 years after radiotherapy but the tumour had reached a considerable volume with extensive bone destruction and multiple cranial nerve palsy [43]. Patients underwent TS/endoscopic (*n* = 7), TC (*n* = 4) or undetermined (*n* = 2) surgery, and frequent post-operative radiotherapy (*n* = 8). Follow-up was available in 8 cases, 6 patients died within 4 years, in one case from early post-operative meningitis [43].

## Discussion

This is the first systematic review on salivary diseases and neoplasia localized to the sellar region. The puzzling case of an apparently intrasellar NNESG removed during TS surgery prompted us to further analyse these conditions, which are not mentioned in exhaustive reviews on sellar/parasellar lesions [47, 48] or single-center experiences reporting rare sellar-suprasellar masses [47, 49, 50]. Indeed, nearly half of the reports were published in the last decade (17/35 papers), in particular those concerning NNESG (7/11 papers, 11/15 cases) and benign primary eST (4/5 papers, 4/7 cases). Strikingly, pre-operative neuroimaging was inconclusive or misleading in all cases. The heterogeneity of radiological descriptions, in part reflecting a variety of pathological histotypes [51], confirms the lack of strongly suggestive features, although cystic components of variable protein content were frequent in NNESG. Where present, DI or rapidly evolving symptoms may help to distinguish such conditions from non-secreting Pit-NETs, but usually suggest alternative diagnosis (craniopharyngiomas, hypophysitis or metastasis). Thus, similar to other rare lesions coming up as pathological surprises [52], they are extremely difficult to consider at the time of pre-operative evaluation. Of note, the pathological diagnosis may also be inaccurate at first observation.

Salivary rests in the pituitary are relatively common incidental findings at autopsy (3.4–8.8%) [10, 11]. This may be explained by pre-existing seromucous glands from the primitive oral cavity remaining in the Rathke’s pouch during migration and persisting during postnatal life [17], similar to ectopic pituitary tissue reported at various locations along its migratory path, including the roof of the nasopharynx [53]. Experimental studies also suggest that Rathke’s pouch components may occasionally differentiate into salivary and adenohypophyseal tissues during organogenesis [54] and that parotid gland tissue may trans-differentiate into pituitary hormone-producing cells under the influence of hypothalamic factors [55]. Embryological development would thus explain why NNESG are found close to the posterior pituitary lobe and sometimes within RCC’s wall [16–18]. In this latter case, the role of salivary remnants in the development of clinical symptoms is not always clear-cut, and some may be incidental findings in the setting of a symptomatic RCC rather than true NNESG [17]. Similarly, we recently observed incidental salivary rests adjacent to an apoplectic gonadotroph PitNET (Suppl Fig. 1), although in our experience this is extremely rare. Alternatively, active secretion from ectopic salivary rests within the cyst was proposed to contribute to RCC enlargement, possibly triggered by parasympathic innervation [18].

The mechanisms leading to NNESG are not fully elucidated but the development of mucinous cysts [14, 18, 19] and/or chronic inflammation [12, 19] are frequently observed. In normal conditions, the lack of neuro-vegetative innervation of the posterior pituitary may prevent the secretion of mucinous material, or local lymphatic/venous reabsorption may remove secretions [20]. Symptomatic NNESG may grow up into the opto-chiasmatic cistern, but exceptionally reach considerable dimensions or appear invasive [16]. Because they more frequently affect young (73.3%) and/or female patients (80%), who present with headache and symptomatic hyperprolactinemia [12–20], NNESG may represent a rare differential diagnosis of cystic sellar lesions in such patients. Hyperprolactinemia likely results from functional disruption of the physiological dopaminergic inhibition, although direct stimulation by EGF, which is abundantly produced by SG, may be hypothesized [56, 57]. This explains why hyperprolactinemia is moderate and promptly normalized by dopamine-agonists in the absence of tumour shrinkage [12, 20]. Less frequently, pre-operative DI or hypopituitarism are present—including growth retardation [15]—and require appropriate hormone replacement therapy. In our patient, the lateral localization of the lesion associated with post-operative recurrence of hyperprolactinemia lead us to reconsider our first diagnosis of intrasellar NNESG with surgical aspiration of prolactinoma cells escaping pathological examination. Furthermore, we found no previous observation of NNESG coexisting with a PitNET. Based on careful revision of serial pre-operative MRI, transient evidence of an intrasphenoidal cyst was noticed, placed on the TS route to the prolactinoma. As only pathological minor salivary glands are seen by radiological imaging [58, 59], we finally concluded for residual inflammation in a submucosal SG following spontaneous reabsorption of the cyst. This pitfall should therefore be considered in the presence of SG acini contaminating surgical fragments obtained by TS, as it may open the possibility of a second TS approach. Once made a definitive diagnosis of NNESG, recurrences have been exceptionally reported [28]. In such cases, a neoplastic origin may not be totally ruled out [28].

ST involving the sellar region have been reported more frequently than NNESG. Patients were adults of any age, mass effects were almost invariably present and amenorrhea–galactorrhea or hyperprolactinemia were occasionally reported. Malignant forms were characterized by a mild female predominance and a major frequency of ocular palsy and symptoms suggestive of hypopituitarism or DI. Similar to primary eutopic ST, they include a variety of histotypes [51, 58], and a minority of eST were found in association with or close to a RCC [7, 33].

Primary benign sellar eST were mostly represented by pleomorphic adenomas. Where specified, they presented as medium/large-sized heterogeneous sellar/suprasellar masses, sometimes with intratumoural hemorrhage or calcifications, potentially mimicking PA, craniopharyngioma, teratoma or chordoma. Hypopituitarism and DI were frequently documented in this group. Post-operative radiotherapy was often proposed due to the risk of recurrence after incomplete surgical removal, although but delayed regrowth could occur [31]. Long-term follow-up is therefore recommended.

Malignant ST usually presented as large, heterogeneous, typically invasive sellar/parasellar masses, and rapid progression could suggest pituitary metastasis or other malignancies. They included a variety of histotypes. Adenoid cystic carcinomas (ACC) accounted for nearly 80% of secondary forms versus 25% of primary malignant eST. With a few exceptions, both were diagnosed earlier (4th–5th decade) than common eutopic ACC (5th–7th decade), which are usually slowly growing [51]. This suggests that sellar ACC may have a different natural history. However, further information is needed to compare the prognosis of sellar ST with similar eutopic histotypes. Radical surgical resection followed by radiotherapy was the treatment of choice in most cases, although first-line (chemo-)radiotherapy was also proposed [33, 46]. Incomplete surgical removal favored tumour progression and/or metastatic spread, with a poor response to radiotherapy and chemotherapy [36]. Of note, one patient died 8 days after surgery for unexplained hypotension [33]. As pituitary function was poorly evaluated in these patients, hypopituitarism could be left untreated.

## Conclusion

Sellar/parasellar lesions derived from SG tissues are rare but challenging conditions. An appropriate pathological characterization is essential for a correct multidisciplinary approach, which should consider and treat their frequent endocrine complications. Where required, hormone replacement therapy is essential to improve patient’s quality of life and prevent the risk of acute adrenal insufficiency. In addition to surveillance for the early recognition of ST recurrences or metastasis, life-long endocrinological follow-up is necessary for the presence of permanent dysfunction or after radiotherapy, which may induce delayed hypopituitarism. Multicenter collection and long-term follow-up would be useful to better define disease evolution and optimal clinical management of these unusual conditions.

## Supplementary Information

Below is the link to the electronic supplementary material.

Supplementary file 1 (PDF 335 KB)
