# Supplementary material for: Salivary gland tissues and derived primary and metastatic neoplasms: unusual pitfalls in the work-up of sellar lesions. A systematic review
Source: J Endocrinol Invest. 2021 May 3;44(10):2103–22. doi: 10.1007/s40618-021-01577-6 (PMC8421317; doi:10.1007/s40618-021-01577-6)
Supplement: Supplementary file 1 — (PDF 335 KB) [file 40618_2021_1577_MOESM1_ESM.pdf]

An example of incidental salivary rests adjacent to PitNET apoplexy

A 68-year-old male patient came back to our observation in June 2020 due to acute headache, blurred vision and general fatigue revealing acute apoplexy complicating a clinically non-functioning PitNET. In 2013, he was referred for the evaluation of an incidental and asymptomatic intrasellar PitNET. Pituitary function was normal, with slightly elevated FSH levels (14 mU/l). Regular follow-up was proposed but the patient escaped clinical observation despite a couple of MRI revealed a slow, still asymptomatic, progression of the lesion. TS was performed after the acute episode due to persisting symptoms, pre-operative endocrine evaluation showed partial hypopituitarism with decreased FSH (4 mU/l) and hypogonadism. The pathological study confirmed the presence of an apoplectic gonadotroph PitNET (Chromogranin A and steroidogenic factor 1 (SF1) immunostaining was clearly reduced by necrosis) with hemorrhagic areas and a small area of ectopic salivary gland rests were noticed. Because there was no reason to believe that these latter were involved in patients' symptoms, there were considered as incidental findings. Here are some representative images of this case:

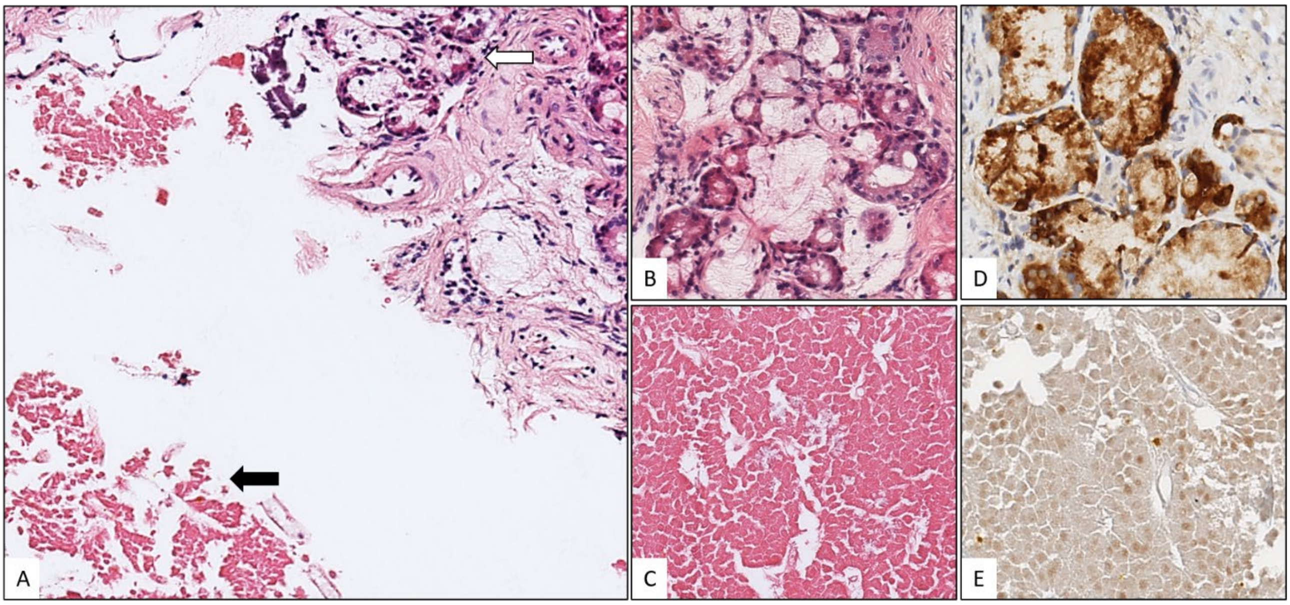

Legend: (A) Optic microscopy (hematoxylin-eosin – 10X HPF) showing glandular structures of mixed type (serous and mucinous) surrounded by fibrous tissue (white arrow) and ghost of tumour cells (black arrow). Detail of glandular structures (B) and dead neuroendocrine pituitary tumour cells (C) in a perivascular arrangement (hematoxylin-eosin – 20X HPF). Immunohistochemical staining was clearly positive for lysozyme in salivary rests (D) and weaker for steroidogenic factor 1 (SF1) in the nuclei of gonadotroph tumour cells, respectively (20X HPF).
